# Study on intracellular delivery of liposome encapsulated quantum dots using advanced fluorescence microscopy

**DOI:** 10.1038/s41598-019-46732-5

**Published:** 2019-07-19

**Authors:** Kristina Bruun, Carsten Hille

**Affiliations:** 1 Physical Chemistry, Institute of Chemistry, University of Potsdam, 14476 Potsdam, Germany; 2 Present Address: Technical University of Applied Sciences Wildau, Hochschulring 1, 15745 Wildau, Germany

**Keywords:** Fluorescence imaging, Drug delivery

## Abstract

Quantum dots increasingly gain popularity for *in vivo* applications. However, their delivery and accumulation into cells can be challenging and there is still lack of detailed information. Thereby, the application of advanced fluorescence techniques can expand the portfolio of useful parameters for a more comprehensive evaluation. Here, we encapsulated hydrophilic quantum dots into liposomes for studying cellular uptake of these so-called lipodots into living cells. First, we investigated photophysical properties of free quantum dots and lipodots observing changes in the fluorescence decay time and translational diffusion behaviour. In comparison to empty liposomes, lipodots exhibited an altered zeta potential, whereas their hydrodynamic size did not change. Fluorescence lifetime imaging microscopy (FLIM) and fluorescence correlation spectroscopy (FCS), both combined with two-photon excitation (2P), were used to investigate the interaction behaviour of lipodots with an insect epithelial tissue. In contrast to the application of free quantum dots, their successful delivery into the cytosol of salivary gland duct cells could be observed when applying lipodots. Lipodots with different lipid compositions and surface charges did not result in considerable differences in the intracellular labelling pattern, luminescence decay time and diffusion behaviour. However, quantum dot degradation after intracellular accumulation could be assumed from reduced luminescence decay times and blue-shifted luminescence signals. In addition to single diffusing quantum dots, possible intracellular clustering of quantum dots could be assumed from increased diffusion times. Thus, by using a simple and manageable liposome carrier system, 2P-FLIM and 2P-FCS recording protocols could be tested, which are promising for investigating the fate of quantum dots during cellular interaction.

## Introduction

During the last decades, the biomedical application of nanomaterials has rapidly increased, especially in the fields of targeted drug delivery, cancer treatment and therapeutics^1–3^. Photoluminescent semiconductor nanocrystals, also known as quantum dots, are outstanding labels for biomedical applications due to their unique optical characteristics that enable them to overcome the limitations of other luminescent probes, such as organic fluorophores^4^. Quantum dots exhibit high molar extinction coefficients with broad excitation spectra and size-tuneable, narrow emission spectra with high luminescence quantum yields and low photobleaching rates^5,6^. Quantum dots are usually synthesised in organic solvents, so that they have to make water-soluble and biofunctionalised for biomedical applications^7^. Due to surface modifications, quantum dots have been used for a wide range of applications in biological research such as luminescent markers for cellular trafficking^8,9^, membrane dynamics and cellular movements^10,11^, single particle tracking^12^ and multicolour imaging^13,14^ or biomarker sensing^15^.

However, the use of water-soluble quantum dots for intracellular labelling can be very challenging. In cell culture media or physiological buffer solutions, quantum dots can exhibit low stability due to particle aggregation and surface degradation, which leads to lower cellular uptake efficiencies^16–18^. This goes along with the fact that quantum dots also tend to aggregate within the cytosol, being often trapped in organelles such as endosomes and lysosomes^19,20^. Moreover, the use of different surface coatings among them polyethylene glycol, polymers, amine and carboxyl functional groups might be responsible for their *in vivo* toxicity^21^. A smart way to improve the intracellular delivery and to neutralise the cytotoxic effects of quantum dots is their encapsulation into specific carrier systems. Liposomes are simply spherical vesicles consisting of phospholipid bilayers surrounding an aqueous volume^22,23^. Liposomes are thought to be biocompatible as well as biodegradable and due to their structure, they can carry both hydrophilic water-soluble molecules in the aqueous core and hydrophobic compounds in the lipid membrane, as previously shown^24,25^. Thus, liposomes have proven useful for cellular quantum dot delivery *via* encapsulation of quantum dots, either into the bilayer membrane of liposomes or into the liposome interior^26,27^. These so-called lipodots have been also exploited to fabricate potential diagnostic and therapeutic tools by additional encapsulation of anticancer drugs such as doxorubicin or docetaxel^28,29^. Thus, drug-coupled lipodots can function for drug delivery, optical tracking and targeted therapy.

For systematic advances in drug delivery in general and lipodot-cell-interaction in particular, adequate information is required about the spatiotemporal interaction of lipodots with the specific biological system. Confocal fluorescence microscopy has been mainly used to study the cellular uptake of quantum dots and their intracellular fate^19,30–35^. However, fluorescence intensity based recordings can be challenging due to varying intensities as a result of different microenvironments, intracellular aggregation or quenching effects. The application of advanced fluorescence techniques can expand the portfolio of useful parameters for a more comprehensive evaluation of lipodots. Thus, fluorescence lifetime imaging microscopy (FLIM) provides access to the luminescence decay time of a fluorophore. This nanosecond decay time is a unique, intrinsic property of a fluorophore and shows a sensitivity towards changes in the microenvironment^36^. The single molecule technique fluorescence correlation spectroscopy (FCS) allows for the determination of physical parameters like the translational diffusion times of particles as well as their hydrodynamic radii^37^. Although FLIM and FCS can obviously improve the interpretation of lipodot-cell-interactions, these techniques have been only sparsely applied in the past. So, changes in the luminescence decay time or in the diffusion time could proof the encapsulation of quantum dots into liposomes in aqueous solution^38–41^ as well as their long term stability^35^. However, only few FLIM studies are available showing a decrease in the quantum dot luminescence decay time after intracellular accumulation by applying cell culture experiments^42–45^. Thus, FLIM recordings could be successfully used for distinguishing the extra- and intracellular localisation of quantum dots as well as their different intracellular microenvironments due to changed luminescence decay times. Nevertheless, to the best of our knowledge no study is available combing FLIM and FCS recordings for the complementary analysis of cellular quantum dot uptake even in a more complex biological system.

In this study, we for the first time applied both techniques complementary, in order to characterise different lipodot preparations *in vitro*, but also to study their intracellular behaviour in a complex biological system. Particularly, by combining FLIM and FCS with two-photon excitation (2P), live cell imaging could be further promoted due to minimised global photobleaching and cell damage. We encapsulated hydrophilic quantum dots into liposomes of different membrane compositions and surface charges, but without further surface modification. This simple and manageable carrier system allowed for evaluation of its cellular uptake into epithelial cells of salivary glands by using FLIM and FCS.

## Methods

### Material and sample preparation

The neutral lipids 1,2-dioleoyl-*sn*-glycero-3-phosphocholine (DOPC), 1,2-dioleoyl-*sn*-glycero-3-phosphoethanolamine (DOPE) and the anionic lipid 1,2-dioleoyl-*sn-*glycero-3-phospho-L-serine (DOPS) were purchased from Avanti Polar Lipids (Alabaster, USA). Qdot 655 ITK Carboxyl Quantum Dots (QD655) were obtained from Thermo Fisher Scientific (Darmstadt, Germany). Phosphate buffered saline (PBS) was from Sigma-Aldrich (Steinheim, Germany), chloroform and other chemicals were from Carl Roth (Karlsruhe, Germany).

Lipodots were prepared using a thin film hydration method. DOPC:DOPE and DOPC:DOPS (3:1 molar ratio) were dissolved in chloroform and dried in a glass vial under nitrogen stream followed by removal of residual solvent under vacuum for ~3 h. The final concentration of lipids was 1 mM. Multilamellar vesicles were obtained by hydrating the lipid films using PBS (pH 7.0) supplemented with 10 nM QD655. After 60 min incubation at 25 °C, the suspensions were subjected to 15 freeze-thaw cycles in liquid nitrogen with vortexing after each cycle.

To form large unilamellar vesicles, the suspensions were passed 35 times through a mini-extruder (Avanti Polar Lipids, Alabaster, USA) using two-stacked polycarbonate membrane filters with a pore size of 100 nm. The final lipodot preparations were stored in the dark at 4 °C and used for experiments up to 7 days. All *in vitro* experiments were performed in PBS. For the cellular uptake experiments, 100 µL of lipodots in PBS were added to a recording chamber containing the salivary gland tissue in 400 µL physiological saline.

### Absorption and luminescence spectra

Absorption measurements were performed with a Lambda 750UV/VIS spectrometer (Perkin Elmer, Waltham, USA) in the range of 300–700 nm. Luminescence emission spectra (500–800 nm, spectral bandwidth Δλ = 1 nm) were recorded with a FluoroMax 4 (Horiba, Kyoto, Japan) using an excitation wavelength of λ_ex_ = 480 nm. Concentrations were adjusted to avoid inner filter effects (absorption maximum below 0.1).

### Time-resolved luminescence measurements

Time-resolved luminescence measurements were performed by using FluoroMax 4 (Horiba) in a time-correlated single-photon counting (TCSPC) mode. For excitation, a LED source (Horiba) with the excitation wavelength set to λ_ex_ = 371 nm and the repetition rate set to 1 MHz was used. The resulting luminescence signal was detected at an emission wavelength of λ_em_ = 655 nm. The luminescence decay curves were fitted multiexponentially with *α*_i_ being the amplitude of the *i*th component with the corresponding decay time *τ*_i_ (Eq. (1)). Additionally, the intensity-weighted average decay time *τ*_av(int)_, which is calculated according to Eq. (2), was used. The goodness of the fits was judged on the reduced ${\chi }_{R}^{2}$-values and randomly distributed residuals.

1 $$\begin{array}{cccc}I(t) & = & \sum _{i=1}^{n}{\alpha }_{i}\,exp(-\frac{t}{{\tau }_{i}})+{I}_{{background}} & \,{\rm{with}}\,{i}=1...3\end{array}$$

2 $$\begin{array}{cccc}{\tau }_{av(int)} & = & \sum _{i=1}^{n}{\alpha }_{i}{\tau }_{i}^{2}/\sum _{i=1}^{n}\,{\alpha }_{i}{\tau }_{i} & {\rm{with}}\,i=1...3\end{array}$$

### Size distribution and zeta potential

The size distribution of empty liposomes and lipodots was determined by dynamic light scattering (DLS) using a Zetasizer Nano ZS (Malvern Panalytical, Kassel, Germany) equipped with a He-Ne laser source (633 nm). The detection of the scattered light occurred at an angle of 173° for reducing artefacts. The sample temperature was adjusted to 25 °C. The same setup was used to determine the zeta potential. Empty liposomes and lipodots were analysed in PBS at pH 7.4.

### Tissue preparation

Salivary glands of the American cockroach were used, whose rearing and tissue preparation was performed as previously described^46^. In detail, a colony of the American cockroach *P*. *americana* (L.) was reared at 27 °C under light/dark cycle of 12 h:12 h at the Department of Animal Physiology (University of Potsdam). The animals had free access to food and water. Only male adults were used for experiments. Salivary glands were dissected in physiological saline containing 160 mM NaCl, 10 mM KCl, 2 mM CaCl_2_, 2 mM MgCl_2_, 10 mM glucose and 10 mM Tris, pH 7.4 as described previously^47^. Small lobes consisting of several acini with their corresponding branched duct system were examined. Thus, lobes were attached to a glass coverslip of precise thickness (170 ± 5 µm) using the tissue adhesive Vectabond (Axxora, Lörrach, Germany).

### 2P-FLIM recordings

2P-microscopy in combination with FLIM was carried out by using the MicroTime 200 fluorescence lifetime microscope system (PicoQuant, Berlin, Germany). The setup was built up and data acquisition and analysis were performed as previously described for quantitative intracellular Ca^2+^ imaging with 2P-FLIM^46^. Thus, it included an inverted microscope (Olympus IX 71) equipped with an Olympus PlanApo ×100/NA 1.4 oil-immersion objective, on which the recording chamber could be mounted. 2P-excitation was performed with a mode-locked femtosecond fiber laser (C-Fiber A 780; MenloSystem, Martinsried, Germany) operating at the fixed wavelength of 780 nm, 50 MHz pulse repetition rate and ~90 fs pulse width. The near infrared (NIR) laser beam was guided toward the objective *via* the microscope side port by using a dichroic mirror (2P-dichroic 725; Chroma, Fürstenfeldbruck, Germany). For rejection of excitation light in the emission pathway, a shortpass filter was used (SP680 OD4, Edmund Optics, Karlsruhe, Germany). The emitted light was guided through a 100 µm pinhole, split by a dichroic mirror FF605 (AHF Analysentechnik, Tübingen, Germany) into the two detection channels and additionally, filtered by passing through the bandpass filters 514/44 (green channel) and 700/75 (red channel) (AHF Analysentechnik), respectively. Single-photon avalanche diodes (SPCM-AQR-13 and SPCM-CD-2801; Perkin Elmer, Waltham, USA) were used for luminescence detection.

Time-resolved fluorescence image acquisition occurred by raster scanning the objective using a *xy*-piezo-positioner (Physik Instrumente, Karlsruhe, Germany) in the time-correlated single-photon counting (TCSPC) mode by using a PicoHarp 300 device with applied time resolution of 8 ps (PicoQuant). Laser power was adjusted to achieve average photon counting rates ≤10^5^ photons/s and peak rates close to 10^6^ photons/s when recording FLIM images, thus below the maximum counting rate allowed by the TCSPC electronics to avoid pulse pile up. Full frame images of 80 µm × 80 µm were acquired in ~50–60 s, with a pixel dwell time of 2.3 ms. Data acquisition and analysis were performed by the SymPhoTime 64 software version 2.3 (PicoQuant). Briefly, all photons collected in a region of interest were used to calculate a global histogram for quantification of the mean fluorescence decay time. Fluorescence decay analysis occurred by deconvolution fitting. The quality of decay fitting was estimated by randomly distributed residuals and comparatively small ${\chi }_{R}^{2}$-values. Fluorescence intensity images were calculated by integrating all detected photons in every pixel, thereby ignoring the temporal information. The full width at half-maximum (FWHM) of the daily measured instrument response function (IRF) of the 2P-FLIM setup from backscattered excitation light was 220 ± 5 ps (*N* = 30), indicating the detector timing resolution as most critical element. The broadening and temporal shift of the IRF at higher photon count rates can be a potential problem for Perkin Elmer SPAD modules^48^. However, the count rate did not influence the temporal position and the FWHM of the IRF up to count rates of 1.2 × 10^5^ photons/s as applied in all *in vitro* measurements, but did so at higher count rates. For recordings in living cells with higher peak count rates this would be problematic, but it can be neglected statistically because of analysing larger regions of interest.

### 2P-FCS recordings

By using the MicroTime200 system, we also performed FCS experiments to determine the diffusion characteristics of the luminescent probes. For experiments in aqueous solution, we used single-point FCS. In this case, the size of the 2P-excitation volume was daily calibrated using a 100 nm aqueous solution of rhodamine 6G, whose diffusion coefficient was known^49^. The experiments were performed at constant room temperature (ϑ = 22 ± 1 °C) with an excitation power for λ_ex_ = 780 nm set to *P*_av_ ~3.7 mW at the objective’s back aperture. The lipodot preparations were analysed at initial phospholipid and QD655 concentrations of 1 mM and 10 nM, respectively. The SymPhoTime 64 software was used for data acquisition and calculation of the corresponding correlation curve, which was based on a cross-correlation routine using the signal of both photodiodes.

FCS measurements in cells were performed using a line-scan FCS method by collecting fluctuation signals along a continuously scanned line^50^. The line-scan of the laser focus was performed in the *xy*-plane in one direction with a fixed *z* position and a pixel dwell time of 40 ms. The movement of the detection volume was controlled directly with the SymPhoTime 64 software, after the linear scan path has been selected from a previously recorded FLIM image. Depending on the duct system, the length of the line-scans was chosen between 40–60 µm.

The autocorrelation function *G*(*τ*) of free diffusing probes was analysed assuming a three-dimensional Gaussian 2P excitation profile using the equation^51^

3 $$\begin{array}{cccc}G(\tau ) & = & \frac{1}{{N}_{m}}{(1+\frac{8D\tau }{{\omega }_{0}^{2}})}^{-1}{(1+\frac{8D\tau }{{z}_{0}^{2}})}^{-1/2} & {\rm{with}}\,D=\frac{{\omega }_{0}^{2}}{8{\tau }_{D}}\end{array}$$

where *τ* is the lag time, *D* is the diffusion coefficient, *N*_m_ is the mean number of fluorescent particles within the detection volume and *τ*_D_ is the average diffusion time of the fluorescent particles diffusing trough the focal volume, expressed by the lateral radius *ω*_0_ and axial radius *z*_0_. For multi-component, three-dimensional diffusion, the autocorrelation function was expressed using the equation

4 $$\begin{array}{cccc}G(\tau ) & = & \frac{1}{{N}_{p}}\sum _{i=1}^{M}\,{f}_{i}{(1+\frac{8D\tau }{{\omega }_{0}^{2}})}^{-1}{(1+\frac{8D\tau }{{z}_{0}^{2}})}^{-1/2} & {\rm{with}}\,i=1...2\end{array}$$

where *N*_*p*_ is the total number of diffusing particles and *f*_*i*_ is the fractional contribution of each component to the correlation function.

The knowledge of the diffusion coefficient was used to calculate the hydrodynamic particle size, which is twice the hydrodynamic radius *R*_h_, using the Stokes-Einstein equation

5 $${R}_{h}=\frac{{k}_{B}T}{6\pi \eta D}$$

where *k*_B_ is the Boltzmann constant, *T* is the temperature and *η* the viscosity of the surrounding medium. Since quantum dots and lipodots were prepared in PBS, the viscosity of *η* = 2.55 mPa s was used for calculations^40^.

In the case of line-scan FCS, the detection volume was repeatedly scanned in a linear fashion within the cells with a constant velocity. The temporal autocorrelation function *G*(0, *τ*) can be calculated with the equation^52,53^

6 $$\begin{array}{cccc}G(0,\tau ) & = & \frac{1}{C\pi {s}^{2}}(\sqrt{\pi }{\mu }_{i}{\rm{erf}}({\mu }_{i})+{e}^{-{\mu }_{i}^{2}}-1) & {\rm{with}}\,{\mu }_{i}=\frac{s}{\sqrt{{\omega }_{0}^{2}+8D\tau }}\end{array}$$

where *C* is the concentration of the particles and *s* the pixel size.

## Results and Discussion

### Characterisation of liposomes and lipodots

Quantum dots with the emission maximum at 655 nm are ellipsoid shaped, with a Cd/Se core and ZnS shell with a 6 nm (minor axis) and 12 nm (major axis) diameter. QD655 with carboxylic acid surface (negative charged) have a hydrodynamic size of 18 nm in borate solution^18^. Since it is known that the encapsulation of such quantum dots in cationic, fusogenic liposomes is problematic due to the electrostatic interactions between the negatively charged quantum dots and the positively charged lipids of the liposomes^54^, we decided to encapsulate the quantum dots in negatively charged liposomes. Moreover, the use of such lipodots should favour their bioimaging applications due to lower cell damage^55^. For empty DOPC:DOPE-liposomes, DLS measurements resulted in an average hydrodynamic size of 111 ± 1.42 nm (*N* = 3) with a polydispersity index of 0.08 ± 0.01. This did not significantly change after loading with QD655, leading to a size of 103 ± 2.99 nm (*N* = 3) with the same polydispersity index of 0.08 ± 0.01. In the case of DOPC:DOPS lipid composition, empty liposomes had a size of 96 ± 0.53 nm (*N* = 3) with a polydispersity index of 0.07 ± 0.01. DOPC-DOPS-lipodots exhibited a similar size of 97 ± 0.82 nm (*N* = 3) with a polydispersity index of 0.10 ± 0.02. The zeta potential is an indicator of the stability of nanocarriers. Since it is known, that at pH near 7.0 the phospholipid anchor DOPE introduces a negative charge to the liposomal surface^56^, both liposome compositions used in this study should possess a negative charge. Zeta potential measurements revealed that the surface potential of empty liposomes was at −5.49 ± 0.12 mV (*N* = 3) for DOPC:DOPE-liposomes and −17.77 ± 2.05 mV (*N* = 3) for DOPC:DOPS-liposomes. After loading with quantum dots, the zeta potential strongly decreased to values of −13.20 ± 2.10 mV (*N* = 3) and −23.53 ± 1.62 mV (*N* = 3) for DOPC:DOPE-lipodots and DOPC:DOPS-lipodots, respectively. These changes indicated the effective interaction of quantum dots with the liposomes. The obtained values were similar to data reported in the literature^32,33,57^ and indicated that the used lipodot systems were sufficiently stable^58^.

**Figure 1:**
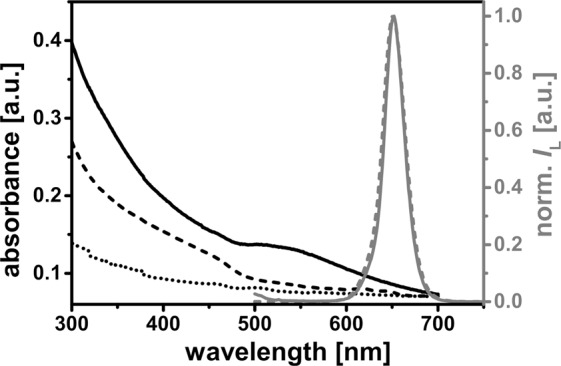
Spectroscopic properties. Absorption spectrum of empty liposomes (DOPC:DOPS) (dotted line) as well as absorption and normalised luminescence spectra (λ_ex_ = 480 nm) of free QD655 (dashed line) and DOPC:DOPS-lipodots (100 nm) (solid line) in phosphate buffered saline (pH 7.0).

The absorption and emission spectra are exemplarily shown for DOPC:DOPS-liposomes in Fig. 1. Since empty liposomes scatter light, their absorption spectrum was just a measure of scattered light with higher intensities at shorter wavelengths as known from literature^59,60^. Free QD655 displayed the attractive property of a broad absorption that gradually increased toward shorter wavelengths, a very common feature for free quantum dots in aqueous solution^61^. In contrast, the absorption spectrum of lipodots was the sum of scatted light from the liposomes and the absorption property of QD655 so that this resulted in a slightly changed absorption spectrum compared to that of free QD655. The luminescence spectrum of lipodots exhibited a symmetric, sharp band with a maximum at 652 nm, very similar to the luminescence spectrum of free QD655. These results suggested that the liposome-encapsulated quantum dots still exhibit a strong luminescence signal without significant quantum dot self-quenching or spectral changes. Moreover, this was a good evidence for the stability of quantum dots within the liposome microenvironment, since blue-shifted emission spectra could indicate quantum dot aggregation or even degradation^61,62^.

### *In vitro* measurements of lipodots luminescence decay times and diffusion times

**Figure 2:**
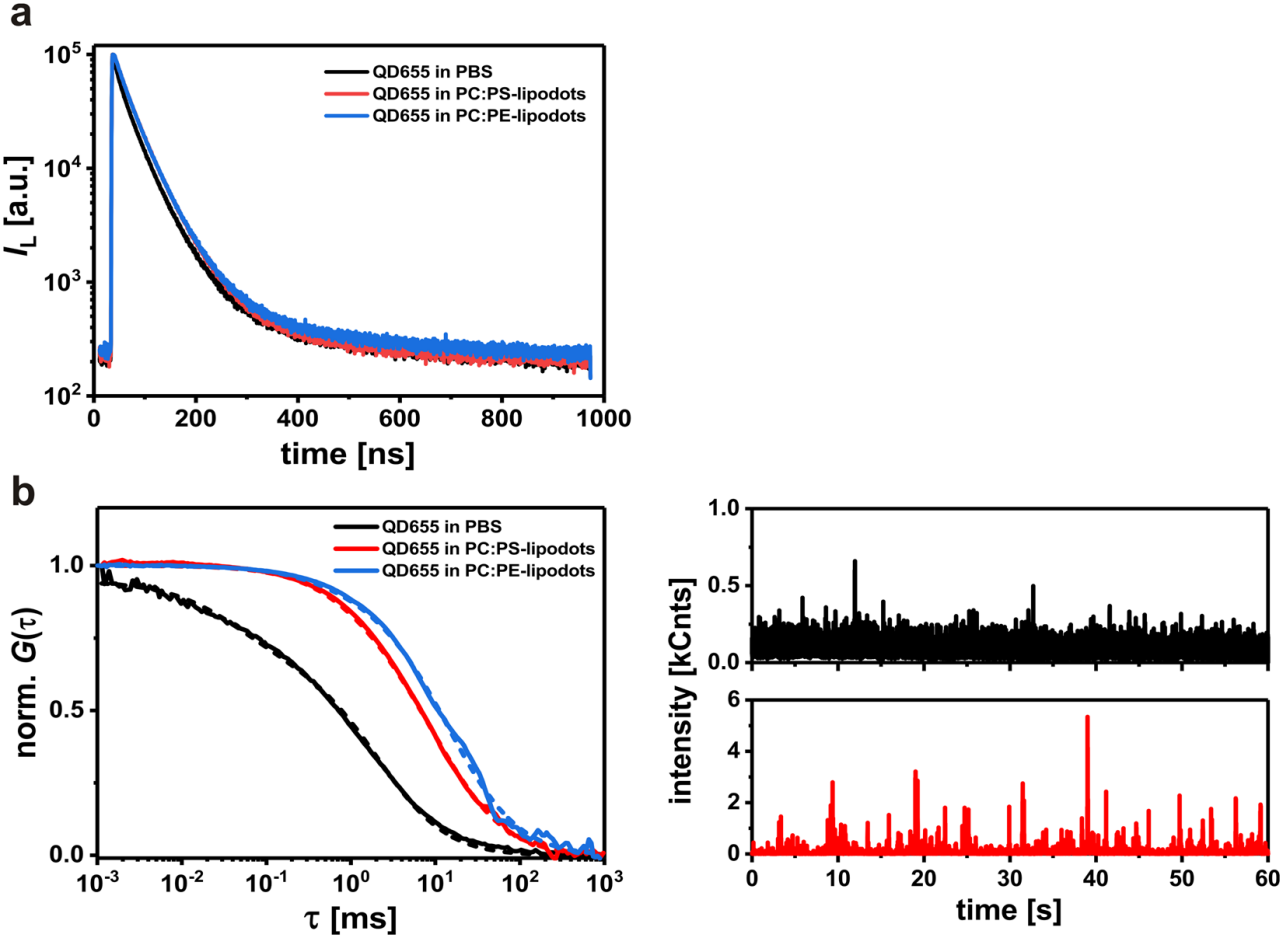
Comparative *in vitro* characterisation of free QD655 and lipodots in phosphate buffer saline (PBS). (**a**) Representative luminescence decay curves of free QD655 (black lines) and lipodots (red & blue lines) recorded at λ_ex_ = 371 nm. (**b**) Left: normalised luminescence autocorrelation curves of free QD655 (black lines) and lipodots (red & blue lines) together with fits (dashed lines) based on a two-component diffusion model according to Eq. (4). Right: corresponding luminescence intensity traces for free QD655 and DOPC:DOPS-lipodots.

In addition to the luminescence intensity of a fluorophore, its luminescence decay time and translational diffusion time can provide additional information about the fluorophores microenvironment. Compared to organic fluorophores and fluorescent proteins, colloidal quantum dots exhibit in general longer luminescence decay times. Their multiexponential decay behaviour is a common feature of quantum dots, although the origin of such complex behaviour is still not well understood. In the present study, we compared the luminescence decay behaviour of free QD655 and lipodots, both dissolved in PBS (Fig. 2a). As expected, for free QD655 a triexponential fit model provided the best fitting results with an intensity-weighted average decay time of *τ*_av(int)_ = 29.7 ± 2.31 ns (${\chi }_{R}^{2}$ = 2.351). In the case of QD655 encapsulated into DOPC:DOPS-liposomes, the intensity-weighted average decay time of the triexponential fit analysis increased to *τ*_av(int)_ = 41.4 ± 2.09 ns (${\chi }_{R}^{2}$ = 1.512), probably indicating a better stabilisation of the quantum dots within the liposomes. A very similar scenario was observed for QD655 encapsulated into DOPC:DOPE-liposomes yielding *τ*_av(int)_ = 39.9 ± 2.71 ns (${\chi }_{R}^{2}$ = 1.495). Generalov *et al*. observed a decrease of the QD655 average luminescence decay time upon encapsulation in phospholipid vesicles^39^, but this was not the case in the present study. This confirmed that the luminescence decay behaviour of quantum dots is strongly dependent on their microenvironment. Nevertheless, their long luminescence decay times allow for straightforward signal separation from background signals such as cellular autofluorescence or scattered light, in comparison to the analysis of quantum dot luminescence intensities.

By using the FCS technique, we analysed intensity fluctuations arising from luminescent particles diffusing through the detection volume yielding their translational diffusion times. Figure 2b shows the normalised autocorrelation curves with their corresponding fitting curves according to Eq. (4) as well as selected luminescence intensity traces for free QD655 and lipodots. For freely diffusing QD655, the diffusion times of *τ*_D1_ = 2.34 ± 0.14 ms (*f*_1_ = 73%) and *τ*_D2_ = 0.04 ± 0.01 ms (*f*_2_ = 27%) were determined. The diffusion time *τ*_D1_ corresponded to the unhindered diffusion of QD655 and fits very well with data from a previous study reporting *τ*_D_ = 2.78 ms for quantum dots in PBS^40^. In comparison with freely diffusing organic dyes such as fluorescein, rhodamine 6G or Atto655-carboxylic acid with sizes in the range of ~0.5 nm, this diffusion is 55 times slower, because of the larger particle size of quantum dots^63,64^. Based on the obtained diffusion coefficient *D*_1_ = 4.5 ± 0.67 µm^2^/s, the hydrodynamic particle size of QD655 was estimated to 35 nm by using the Stokes-Einstein relation (Eq. (5)). The presence of a second diffusion component with the diffusion time *τ*_D2_ was not entirely clear. A possible explanation could be the unstable blinking of quantum dots due to the surface charge density, induced by a pH change from pH 9 in borate buffer to pH 7 in PBS as well as the used excitation light intensities^40,65^.

When QD655 was encapsulated into a liposomes, the observed diffusion behaviour corresponded to the lipodot entity and was significantly slower than in the case of freely diffusing QD655 (Fig. 2b). Thus, the autocorrelation curve of the DOPC:DOPS-lipodots shifted to longer diffusion times yielding *τ*_D1_ = 11.0 ± 0.99 ms (*f*_1_ = 88%) and *τ*_D2_ = 0.27 ± 0.05 ms (*f*_2_ = 12%). Again, a very similar scenario was observed for DOPC:DOPE-lipodots yielding *τ*_D1_ = 11.4 ± 1.71 ms (*f*_1_ = 86%) and *τ*_D2_ = 0.21 ± 0.11 ms (*f*_2_ = 14%). The dominant time *τ*_D1_ could be attributed to the diffusion of lipodots and the minor time *τ*_D2_ to the diffusion of an unknown component. Using the calculated diffusion coefficient *D*_1_ = 1.42 ± 0.22 µm^2^/s, we found for the lipodots a hydrodynamic particle size of 120 nm, which fits very well with the size determined by DLS recordings (see above). Finally, the comparison of the average count rates obtained from luminescence intensity traces clearly indicated the higher luminescence intensity bursts of lipodots compared to that of free QD655. Camblin *et al*. have reported similar diffusion time changes for freely diffusing quantum dots and polymersomes-encapsulated quantum dots being *τ*_D_ = 4 ms and *τ*_D_ = 15–17 ms, respectively^35^.

Although the encapsulation efficiency was not analysed in detail, the complementary luminescence decay time and translational diffusion time recordings allowed for proofing an effective and stable loading of QD655 into both types of liposomes under the applied experimental conditions. First, after the extrusion process during the lipodots preparation procedure quantum dot luminescence could be detected on the membrane filters under UV light. Thus, the quantum dot sticking at the polycarbonate membrane allowed for separation of free QD655 from liposome-encapsulated QD655 as previously reported^66^. Second, the QD655 luminescence decay time increased uniformly in the presence of both types of liposomes (*τ*_av(int)_ ~30 ns *vs*. ~40–41 ns) indicating the changed quantum dot microenvironment after encapsulation. Third, the increase in the diffusion time after lipodots preparation (*τ*_D1_ ~2 ms *vs*. ~11 ms) indicated the recording of lipodots rather than free QD655. In addition, the very dominant relative fraction of this slow diffusion time (*f*_1_ ~86–88%) also supported the sufficient lipodots separation from free QD655. Thus, both types of lipodots preparations seemed to be feasible for cellular imaging studies.

### Autofluorescence of salivary gland duct cells

**Figure 3:**
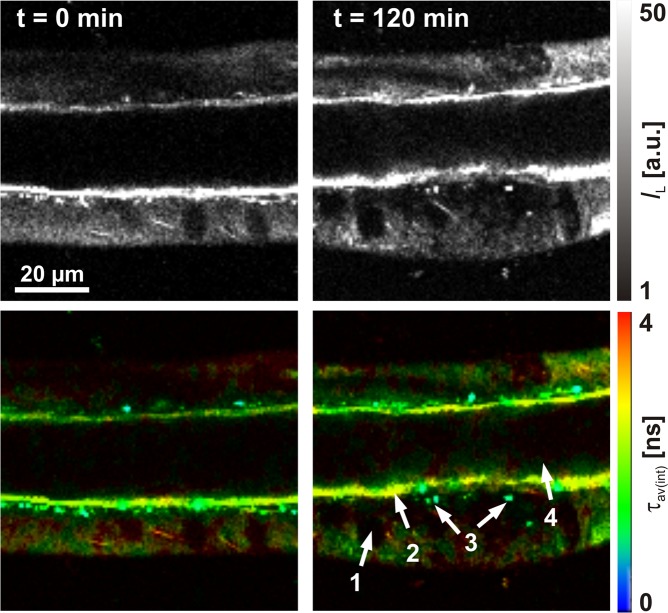
Cellular autofluorescence. Fluorescence intensity images (top) and 2P**-**FLIM images (bottom) of an unstained cockroach salivary duct treated only with physiological saline (pH = 7.4), displaying the autofluorescence (λ_em_ = 492 nm–680 nm) after 2P-excitation at λ_ex_ = 780 nm for 0 min and 120 min, The median optical section plane through the gland duct indicated the following structural features: 1 cell nucleus, 2 luminal cuticle, 3 point-shaped structures, 4 lumen. Recording parameters: 128 pixel × 128 pixel, 80 μm × 80 μm, pixel dwell time 2.3 ms/pixel, TCSPC time resolution 8 ps, laser repetition rate 50 MHz.

For live cell applications, we used salivary glands of the American cockroach, representing a well-established model system for studying transepithelial ion transport processes. In addition, the salivary glands display straightforward methodological accessibility and ease of physiological manipulation^67,68^. Here, we initially determined whether the salivary gland ducts show time-dependent changes, induced by the incubation medium or irradiation of the applied excitation light (*P*_av_ = 3.7 mW, measured at objective back aperture). The morphology of the duct cells before and after treatment with physiological saline (pH = 7.4), similar to that of the lipodots *in vitro* experiments, for 120 min is shown in Fig. 3. The overview images illustrate the median optical section plane through the duct with the prominent lumen, a thin continuous luminal cuticle, numerous cellular nuclei and apically located, characteristic point-shaped structures of yet unknown origin. The unloaded cells displayed a comparatively low autofluorescence when excited at 780 nm as expected for 2P-excitation in the NIR spectral range in comparison to the corresponding 1P-excitation in the blue spectral range. In addition, cells stayed intact over the measurement time without visible cell volume changes or photodamage effects. The autofluorescence mainly resulted from the redox pairs nicotinamide adenine dinucleotide (NADH/NAD^+^) and flavin adenine (FADH_2_/FAD), serving as electron carriers during ATP-producing oxidative phosphorylation^36^. However, only reduced NADH and oxidized FAD are fluorescent and can be monitored by fluorescence microscopy. Both redox pairs exist in two physiological forms, a free form and a protein-bound form, which exhibit well separated fluorescence decay times^69^. Upon binding to mitochondrial proteins, the fluorescence decay time of FAD decreases, whereas that of NADH increases. Thus, to discriminate between free and protein-bound forms, biexponential fluorescence lifetime imaging maps of cellular metabolism are typically generated^70^. From time-resolved measurements we examined for the duct cells a biexponential fluorescence decay behaviour with two distinct decay time components *τ*_1_ = 2.60 ± 0.10 ns (*α*_1_ = 19%) and *τ*_2_ = 0.36 ± 0.01 ns (*α*_2_ = 81%) with an intensity-weighted average decay time *τ*_av(int)_ = 1.77 ± 0.06 ns (*N* = 12). Since 2P-excitation at 780 nm leads to excitation of both, NADH and FAD, the biexponential decay behaviour most probably reflects both species. This result was in good agreement with the fluorescence decay times of NADH and FAD described previously^69^. More important, the measured autofluorescence decayed one order of magnitude faster than that of free or encapsulated QD655 allowing for reliable signal analysis.

Furthermore, by using the line-scan FCS method in the intracellular region we monitored the diffusion of autofluorescent cellular components. While in most cases we observed a lack of autocorrelation curves due to relatively low autofluorescence, in some duct cells we could determine correlations resulting in an average diffusion time of *τ*_D_ = 370 ± 35 ms, which corresponded to an average diffusion coefficient of *D* = 0.071 ± 0.007 µm^2^/s (*N* = 30). This value is about four orders of magnitude smaller than the diffusion coefficient of NADH and FAD measured in aqueous buffer and water, respectively^71,72^. This fact indicates the presence of further autofluorescent species. Moreover, by comparing the autocorrelation amplitudes obtained from the autocorrelation curves at 0 min and 120 min, we observed in most cases an increase in the number of molecules. A similar effect was also described by Brock *et al*. and was attributed to either subcellular motions or to an increase in the fluorescence signals of certain molecules above the detection threshold, due to the metabolism-dependent changes in their quantum yields^73^. Since the diffusion of molecules in the cytoplasm is a complex phenomenon, strongly dependent on the cell physiology, it is difficult to compare the results of different studies in different cell types^51,73,74^. Therefore, comprehensive studies are required in order to correctly assign the measured diffusion time of autofluorescent cellular components.

### Cellular uptake experiments with free QD655

**Figure 4:**
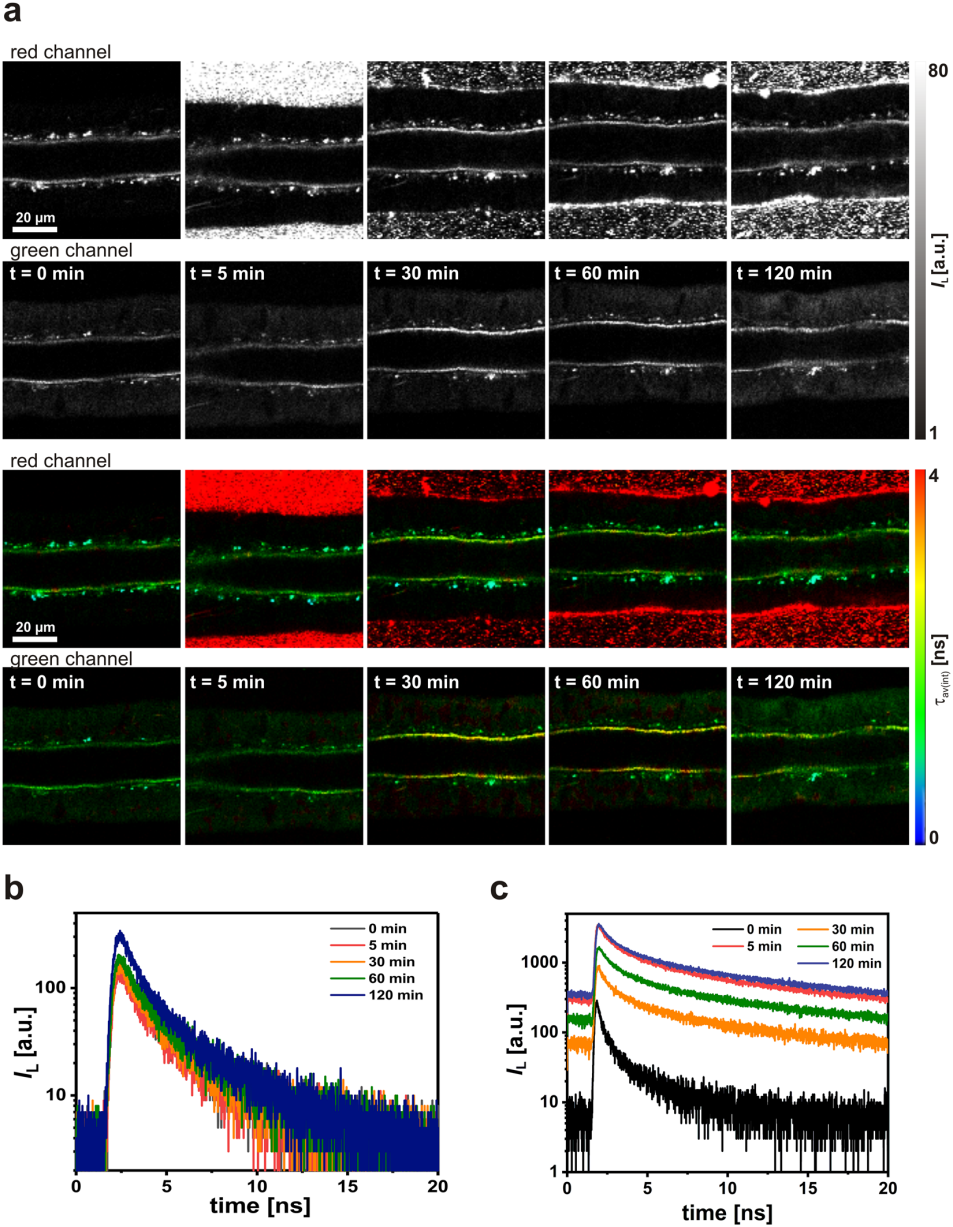
Analysis of the cellular uptake of free QD655 into living duct cells. (**a**) Representative 2P-luminescence intensity images and corresponding 2P-FLIM images of duct cells recorded in the green (BP 514/44) and red (BP 700/75) detection channels at distinct time points. The cells were treated for 120 min with physiological saline containing 5 nm QD655. (**b**) Luminescence decay curves extracted from intracellular regions of interest from 2P-FLIM images recorded in the green detection channel. (**c**) Luminescence decay curves extracted from extracellular regions of interest from 2P-FLIM images recorded in the red detection channel.

In order to evaluate the benefit of lipodots for cellular uptake experiments, we started with incubation experiments using only free QD655. Thus, small salivary gland lobes were treated for 120 min with physiological saline containing 5 nM QD655 and the described results are representative for *N* = 6 independent experiments. Since the cellular autofluorescence mainly resulted from NADH and FAD as discussed above and according to their luminescence emission maxima^36^, the luminescence intensity within the duct cells was more pronounced in the green than in the red detection channel at *t* = 0 min (Fig. 4a). The bath incubation with free QD655 for 120 min did not significantly change the intracellular luminescence intensity in both detection channels. In addition, the weak intracellular luminescence decayed in the range of the measured autofluorescence (Fig. 4b). However, no FCS data could be analysed due to the low luminescence signals. On the other hand, considerable QD655 luminescence could be recognised in the extracellular surrounding physiological saline, but only in the red detection channel as expected from their emission spectrum (see Fig. 1). The luminescence in this channel decayed triexponentially with intensity-weighted average decay times of *τ*_av(int)_ = 5.8–6.3 ns at the different time points (Fig. 4c). These luminescence decay times were shorter than that of free QD655 in pure PBS (see Fig. 2). This could be probably due to the unspecific interaction with extracellular components as well as the limited time resolution of the 2P-FLIM imaging setup compared to the *in vitro* spectroscopic setup. Thus, the applied free QD655 could be not sufficiently incorporated into the duct cells and were mainly accumulated in the cellular surrounding due to sedimentation at the coverslip bottom of the recording chamber. Similar reports on low cellular uptake efficiencies of free, non-functionalised quantum dots have been published previously^16–18^.

### Cellular uptake experiments with lipodots

**Figure 5:**
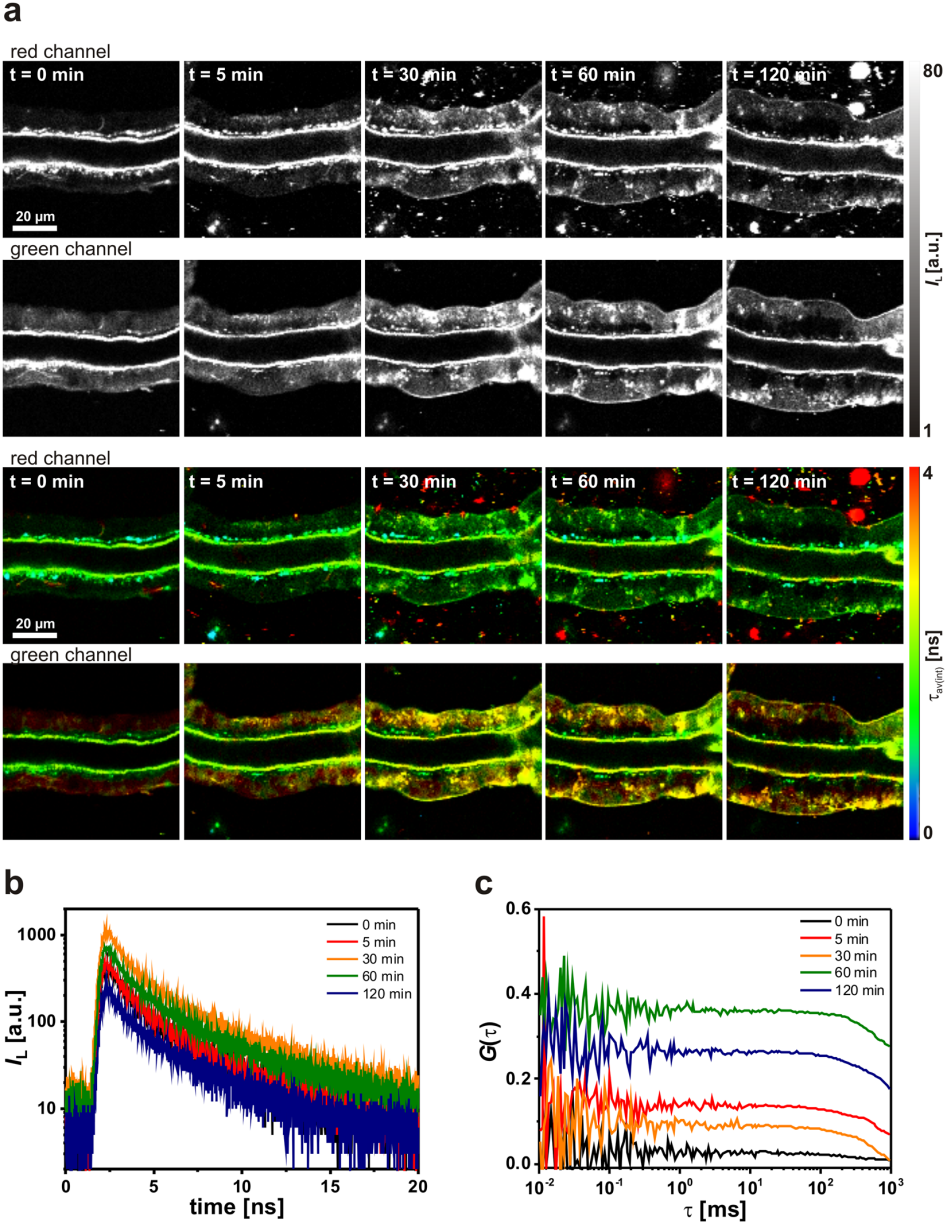
Analysis of the cellular uptake of neutral lipodots into living duct cells. (**a**) Representative 2P-luminescence intensity images and corresponding 2P-FLIM images of duct cells recorded in the green (BP 514/44) and red (BP 700/75) detection channels at distinct time points. The cells were treated for 120 min with physiological saline containing 10 nM DOPC:DOPE-lipodots. (**b**) Luminescence decay curves extracted from intracellular regions of interest from 2P-FLIM images recorded in the green detection channel. (**c**) Autocorrelation curves obtained from 2P-FCS line-scan measurements performed in duct cells recorded in the green detection channel.

Next, we focused on the potential of lipodots for cellular uptake into salivary gland duct cells. In incubation experiments similar to that performed with free QD655, small salivary gland lobes were treated for 120 min with physiological saline containing 10 nM lipodots. Prior lipodots addition, only the autofluorescence signal could be detected, as mentioned above. However, in the presence of lipodots the acquired luminescence intensity images and the corresponding 2P-FLIM images suggested a successful uptake of QD655 from the DOPC:DOPE-lipodots (Fig. 5a). Since, no visible changes of the cellular morphology between lipodots-treated cells and the control untreated cells were observed, we assumed no significant cytotoxicity of QD655 in the used cells. Approximately 5 min after incubation, we observed inhomogeneously distributed bright luminescent spots within the cytosol, but excluded from the nucleus. The luminescence intensity in the cells slightly increased with acquisition time up to a saturation point at approx. 30 min. A similar uptake pattern has been reported for QD610-loaded liposomes^33^ as well as for QD655 (PEG)-loaded liposomes^39^. The intracellular luminescent spots could be attributed to aggregates of quantum dots formed intracellularly^19,20,75,76^. Surprisingly, this luminescence signal was more pronounced in the green detection channel than in the red detection channel, although the QD655 emission maximum was observed at 652 nm (see Fig. 1). This in turn could by partly explained by luminescence quenching of intact quantum dots due to the changed intracellular microenvironment, especially concerning the ionic strength and possible interactions of quantum dots with cellular molecules^61,76^. In addition, the enhanced signal in the green detection channel indicated a blue shift in the quantum dot luminescence. This could be in fact the result of degradation processes after release of QD655 from lipodots for instance by endosomal and lysosomal activity. Moreover, we observed also an increasing clustering of luminescent particles in the extracellular surrounding medium without any changes in the red luminescence (bright red spots only in the red detection channel). This clustering behaviour of lipodots in physiological saline is different to that observed in the case of free QD655 without any liposomal carrier (see Fig. 4). The assumed effect of quenching and degradation of quantum dot luminescence in the cells was confirmed by the luminescence decay curves obtained from the regions of interest in the green detection channel, which were fitted to a biexponential decay function yielding reasonable residuals and reduced ${\chi }_{R}^{2}$ values (Fig. 5b). Thus, the resulted average decay times were one order of magnitude shorter than those of free QD655 and lipodots in PBS microenvironment (see Fig. 2a). After a saturation point, reached approximately after 30 min incubation, the intensity-weighted average decay time *τ*_av(int)_ continued to decrease from 2.70 ± 0.29 ns at 30 min up to 2.57 ± 0.38 ns at 120 min (*N* = 18) measured in the green detection channel. Thus, the observation of strong blue-shifted luminescence emission combined with shorter luminescence decay times most probably indicates the intracellular release of free QD655 rather than accumulation of intact lipodots.

Furthermore, we examined the diffusion behaviour of quantum dots in cells by performing line-scan FCS in the cellular regions. The cytoplasm is a heterogeneous environment consisting of internal membrane structures, cytoskeletal meshwork and other macrobiomolecules, so that the diffusive transport of molecules deviates from ideal behaviour and strongly depends on the position within the cell^77^. It has been already reported, that while most of quantum dot aggregates in living cells are immobile, some of them move, often in a fairly straight paths in a rapidly fashion^76^. However, Ruan *et al*. postulated a random diffusion of released quantum dots in crowded cytoplasm. They estimated that the time to diffuse through a 20 µm-diameter cell for a single quantum dot of a hydrodynamic radius of 10 nm would be ~30 s, whereas it would be ≥5 h for a vesicle of a hydrodynamic size of 80 nm^30^. However, in most cases, we could measure reasonable autocorrelation curves, that showed in general a shift to slightly longer diffusion times compared to the autocorrelation curves obtained for the intrinsic autofluorescence alone (Fig. 5c). While for some autocorrelation curves the diffusion times increased up to *τ*_D_ = 1.3 ± 0.2 s (*N* = 12), in several cases we determined two different diffusion times with *τ*_D1_ = 800 ± 113 ms (*f*_1_ = 61%) and *τ*_D2_ = 1.17 ± 0.52 ms (*f*_1_ = 39%) (*N* = 26). We compared these results with the diffusion times of freely diffusing QD655 in PBS and we attributed the slow diffusion component to possible intracellular quantum dots aggregates, as mentioned before. The appearance of the faster diffusion time component could be the result from single diffusing free quantum dots, even though this value is smaller than that obtained from free QD655 in PBS (see Fig. 2b). The discrepancy is probably based on the fitting procedure of the quite different time components (800 ms *vs*. 1.17 ms) leading to an underestimation of the fast time component. This fact would support a successful release of quantum dots from the lipodots. Previous studies suggest that especially endocytosis and lipid-mediated fusion with the cell membranes are the major pathways for liposomal internalisation^78^. Dudu *et al*. revealed a consistent difference in the mechanism of quantum dot internalisation in two different cancer cell lines using the same cationic liposomes^31^. Since it is known, that the liposome-binding with the cell plasma membrane is non-specific and mostly driven by electrostatic interactions between the liposome carriers and proteoglycans present at the cell surface, the fusion probability of the liposomes strongly depends on their surface charge and lipid composition^31,79^. It has been proposed that the use of cationic lipids is essential for increased cellular uptake of liposomes^32,54,80^. Recently, it has been shown that in addition to neutral and cationic lipids the presence of an aromatic molecule significantly improves the fusion efficiency of liposomes^81^. However, in this study the presence of neutral DOPE in the lipodots was sufficient and could be reasonable for their membrane association. DOPE facilitates the internalisation by fusion with the membrane, which can be attributed to their minimally hydrated head group and the resulting high affinity toward cell membrane^82^. This is called the transbilayer internalisation pathway^83^. Nevertheless, further studies have to be performed in future to investigate the mechanisms of cellular uptake as well as intracellular trafficking mechanism for lipodots, especially in different complex cell types.

**Figure 6:**
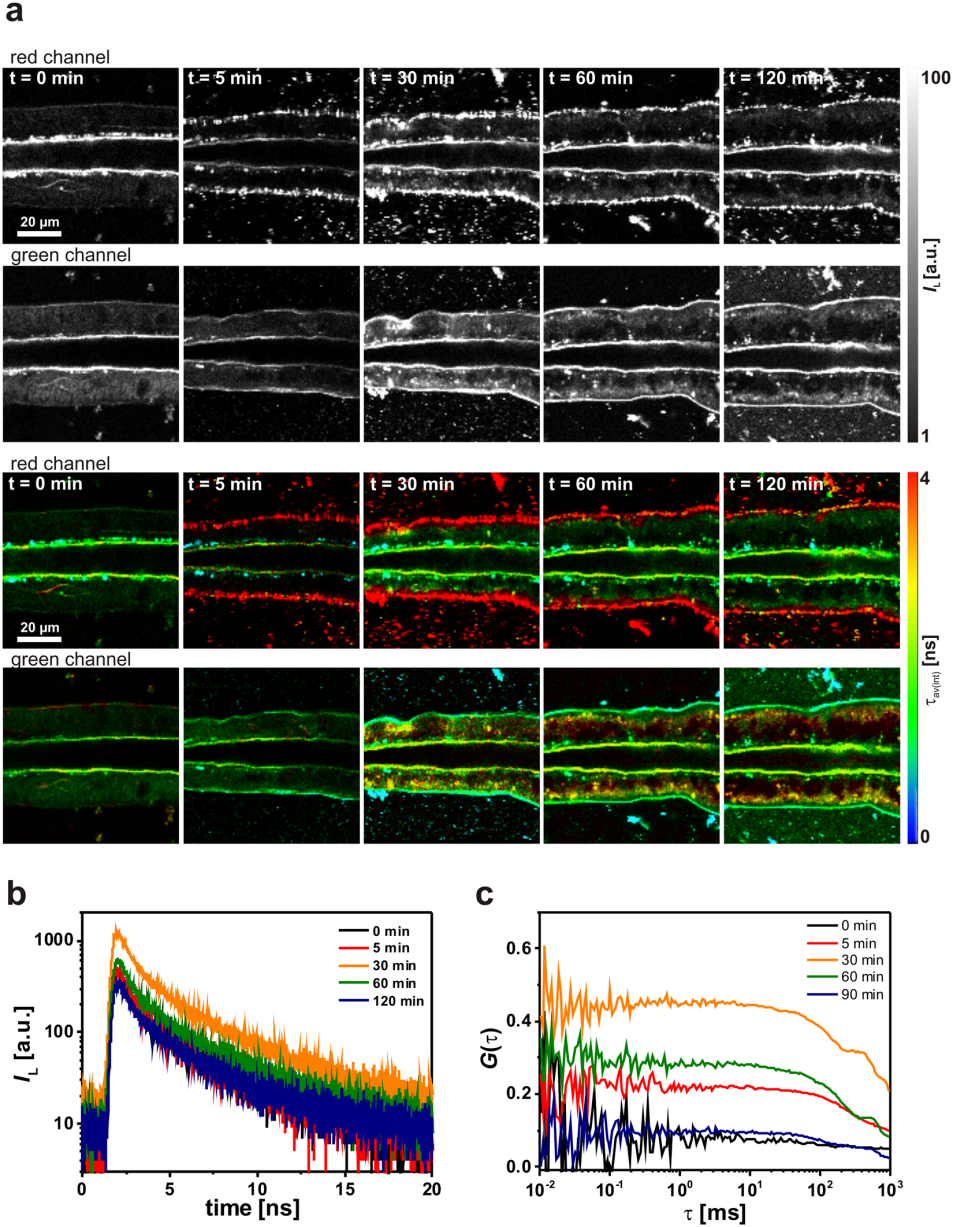
Analysis of the cellular uptake of negatively charged lipodots into living duct cells. (**a**) Representative 2P-luminescence intensity images and corresponding 2P-FLIM images of duct cells recorded in the green (BP 514/44) and red (BP 700/75) detection channels at distinct time points. The cells were treated for 120 min with physiological saline containing 10 nM DOPC:DOPS-lipodots. (**b**) Luminescence decay curves extracted from intracellular regions of interest from the 2P-FLIM images recorded in the green detection channel. **(c)** Autocorrelation curves obtained from 2P-FCS line-scan measurements performed in duct cells recorded in the green detection channel.

In addition, we tested the interactions of DOPC:DOPS-lipodots with salivary gland duct cells. Since DOPC:DOPS-lipodots showed a more negative zeta potential compared to DOPC:DOPE-lipodots, they could exhibit a different interaction behaviour. The 2P-FLIM images of the duct cells exposed to the negatively charged lipodots showed in addition to cytoplasmic luminescence also a strong luminescence signal at the membrane, indicating an accumulation of lipodots at the cell surface (Fig. 6a). This was especially true for the red detection channel, monitoring the spectroscopic unchanged quantum dots. As soon as QD655 was accumulated intracellularly, the luminescence decay time decreased and the emission was blue-shifted (green detection channel). Thus, the intracellular quantum dot stability seems to be insufficient and further quantum dot surface modifications are required. Nevertheless, no considerable differences in the intracellular labelling pattern, luminescence decay time and diffusion behaviour could be observed between both types of lipodots. Indeed, also in the case of DOPC:DOPS-lipodots a decrease in the intensity-weighted average decay time from *τ*_av(int)_ = 2.58 ± 0.37 ns at 30 min up to *τ*_av(int)_ = 2.20 ± 0.24 ns at 120 min (*N* = 13) has been observed (Fig. 6b). A shift of the autocorrelation curves to longer diffusion times (Fig. 6c) resulted either in a single diffusion time of *τ*_D_ = 792 ± 175 ms (*N* = 18) or two different diffusion time components with *τ*_D1_ = 523 ± 107 ms (*f*_1_ = 55%) and *τ*_D2_ = 1.85 ± 0.96 ms (*f*_1_ = 45%) (*N* = 10).

**Figure 7:**
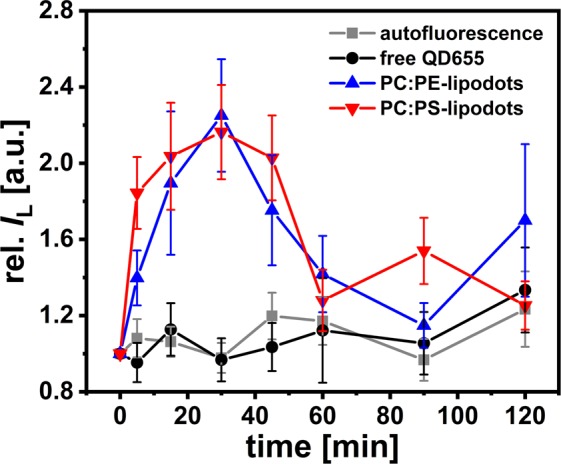
Time-dependent relative luminescence intensity changes in duct cells. Intracellular luminescence intensity changes of regions of interest were recorded during a period of 2 h for cells in physiological saline alone (grey line) and separately for cells incubated in physiological saline with 5 nM QD655 (black line) or 10 nM lipodots using one of the two different lipid compositions (red & blue lines). Intensity changes relative to the starting point (*t* = 0) were analysed from 2P-FLIM images recorded in the green detection channel; means ± SEM of *N* = 3–18.

Figure 7 shows the time-dependent changes in the intracellular luminescence intensity measured in the green detection channel for the two differently composed lipodots compared with free QD655. The application of free QD655 did not result in significant changes compared to the intracellular autofluorescene, indicating their ineffective cellular uptake. On the other hand, the application of encapsulated quantum dots resulted in their sufficient intracellular uptake, however, accompanied with altered luminescence properties. An increase in the luminescence intensity was observed after the initial 5 min bath application of lipodots to the cells, but this change was surprisingly reversible. After approx. 30 min incubation period, the intracellular luminescence intensity slowly decreased, probably due to intracellular degradation. This cellular uptake behaviour was consistent with the findings for two different cell lines, HeLa and GPC^PDGF^, which showed a rapid uptake of cationic lipodots, although the intracellular luminescence intensity increased continuously, even after 60 min incubation period^31^. Internalisation of cationic liposomes labelled with quantum dots into human lung epithelial carcinoma A549 cells has been already observed after 5 min post-injection^32^, whereas in the case of anionic lipodots a luminescence signal in melanoma cells could be only observed after 2 h incubation period^33^. That study pointed out, that the internalisation efficiency of quantum dot-loaded liposomes strongly depends on the liposomal cargo and composition as well as the cell types, so it is often difficult to compare the results among each other. However, more systematic work needs to be carried out to reveal and interpret the exact intracellular trafficking mechanism for lipodots. Furthermore, to improve the biological application of quantum dots, novel forms of *in vivo* tracking, including multi-colour and three-dimensional quantum dot tracking tools are essential^84^. In recent years, a new class of nanocrystal quantum dots, the so called non-blinking giant quantum dots, has been introduced^85^. The stable emission from these quantum dots allows an extended tracking duration compared to conventional core/shell blinking quantum dots, leading to observations of heterogeneous receptor diffusion occurring over time scales of minutes^86^.

## Conclusion

In this study, we for the first time characterised lipodots *in vitro* and investigated their cellular uptake into epithelial cells of a salivary gland tissue by using FLIM and FCS technique complementarily, each combined with 2P-excitation. Thus, the obtained parameters allowed for the successful characterisation of lipdot-cell-interactions (Table 1). With access to the microenvironment-dependent luminescence decay times and the size-dependent translational diffusion times of luminescent particles such as quantum dots, one can evaluate their encapsulation into or their release from a carrier system as well as their extra- or intracellular localisation. In combination with temporally and spectrally resolved image acquisition this can obviously improve the interpretation of lipodot-cell-interactions. Here, we encapsulated QD655 into liposomes of two different lipid compositions and surface charges. The prepared lipodots were characterised *in vitro* and could be successfully applied for the cellular uptake experiments. Instead of isolated cells from cell culture, we used a living gland tissue preparation possessing cell junctions and an extracellular matrix as additional potential barriers making quantum dot uptake experiments more realistic. The lipodots showed a fast accumulation at the membrane surface. After 5-minute incubation period, we observed a cellular uptake and time-dependent changes in accumulation and intracellular distribution of quantum dots in the cytosol of the duct cells independent of the lipid composition. Luminescence decay time measurements confirmed luminescence quenching of the quantum dots, probably due to changed microenvironment and interaction of these nanoparticles with intracellular molecules. Furthermore, FCS data analysis from intracellular regions revealed diffusion of two different components, which could be attributed to possible intracellular clustering of the quantum dots and single diffusing quantum dots, respectively. By using a simple and manageable liposome carrier system, 2P-FLIM and 2P-FCS recordings can lead to new information about the stability and localisation of quantum dots within a complex biological microenvironment. This technical toolbox allows for further quantum dot-cell-interaction studies and spatiotemporal drug carrier-cell-interaction in general. Here, intact quantum dots at the outer cellular membrane and somehow degraded quantum dots within the living cells could be distinguished. However, additional experiments can be performed now, addressing the intracellular uptake and chemical modification mechanisms of lipodots. In this context, the application of functionalised liposome carrier systems is conceivable for more specific biological and clinical applications. Thereby, enhanced targeted delivery can be obtained with immunoliposomes consisting of surface-attached antibodies or their fragments and more stable, long-circulating liposomes can be realised by coating with polymers such as PEG^87,88^.

**Table 1:** Summary of the obtained main parameters for the characterisation of lipdot-cell-interactions.

Sample	Particle size/nm^a^	Luminescence intensity^b^	Luminescence decay time *τ*_av(int)_/ns^c^	Translational diffusion time *τ*_D_/ms (fractional contribution *f*_i_/%)^d^	Diffusion coefficient *D*/µm^2^ × s^−1^ (hydro-dynamic radius *r*_h_/nm)
Free QD655		λ_em_ = 655 nm	29.7 ± 2.31	2.34 ± 0.14 (73) 0.04 ± 0.01 (27)	4.5 ± 0.67 (35)
***Empty liposomes***:					
DOPC:DOPS	96 ± 0.53				
DOPC:DOPE	111 ± 1.42				
***Lipodots***:					
DOPC:DOPS	97 ± 0.82	λ_em_ = 652 nm	41.4 ± 2.09	11.0 ± 0.99 (88) 0.27 ± 0.05 (12)	1.4 ± 0.22 (120)
DOPC:DOPE	103 ± 2.99	λ_em_ = 652 nm	39.9 ± 2.71	11.4 ± 1.71 (86) 0.21 ± 0.11 (14)	1.4 ± 0.22 (120)
Untreated cells^e^		weak	1.77 ± 0.06	370 ± 35 (100)^f^	0.071 ± 0.007
***Treated cells***^e^:					
Free QD655		weak			
DOPC:DOPS-lipodots		*I*_L,green_ > *I*_L,red_	2.58 ± 0.37^g^	523 ± 107 (55) 1.85 ± 0.96 (45)	
DOPC:DOPE-lipodots		*I*_L,green_ > *I*_L,red_	2.70 ± 0.29^g^	800 ± 113 (61) 1.17 ± 0.52 (39)	

^a^Data from DLS recordings.

^b^*I*_L,red_ and *I*_L,green_ correspond to the luminescence intensities in the red and green detection channel, respectively.

^c^Data from FLIM recordings, shown as intensity-weighted average decay times.

^d^Data from FCS recordings.

^e^Intracellular luminescence.

^f^Weak signal.

^g^Data after 30 min incubation.

## Data Availability

All relevant data are included in this published article.

## References

[1] Farokhzad OC, Langer R (2009). Impact of nanotechnology on drug delivery. ACS Nano.

[2] Babu A, Templeton AK, Munshi A, Ramesh R (2014). Nanodrug delivery cystems: a promising technology for detection, diagnosis, and treatment of cancer. AAPS Pharm. Sci. Tech..

[3] Jahangirian H, Lemraski GE, Webster TJ, Rafiee-Moghaddam R, Abdollahi Y (2017). A review of drug delivery systems based on nanotechnology and green chemistry: green nanomedicine. Int. J. Nanomedicine.

[4] Zhou J, Yang Y, Zhang C (2015). Toward Biocompatible Semiconductor Quantum Dots: From Biosynthesis and Bioconjugation to Biomedical Application. Chem. Rev..

[5] Kippeny T, Swafford LA, Rosenthal SJ (2002). Semiconductor nanocrystals: a powerful visual aid for introducing the particle in a box. J. Chem. Edu..

[6] Smith AM, Duan H, Mohs AM, Nie S (2008). Bioconjugated quantum dots for *in vivo* molecular and cellular imaging. Adv. Drug Deliv. Rev..

[7] Foubert A (2016). Bioconjugation of quantum dots: Review &amp; impact on future application. TrAC Trends Anal. Chem..

[8] Alivisatos AP, Gu W, Larabell C (2005). Quantum dots as cellular probes. Annu. Rev. Biomed. Eng..

[9] Rajan SS, Liu HY, Vu TQ (2008). Ligand-bound quantum dot probes for studying the molecular scale dynamics of receptor endocytic trafficking in live cells. ACS Nano.

[10] Chen H, Titushkin I, Stroscio M, Cho M (2007). Altered membrane dynamics of quantum dot-conjugated integrins during osteogenic differentiation of human bone marrow derived progenitor cells. Biophys. J..

[11] Gonda K, Watanabe TM, Ohuchi N, Higuchi H (2010). *In vivo* nano-imaging of membrane dynamics in metastatic tumor cells using quantum dots. J. Biol. Chem..

[12] Levi V, Gratton E (2007). Exploring dynamics in living cells by tracking single particles. Cell Biochem. Biophys..

[13] Gao X, Cui Y, Levenson RM, Chung LWK, Nie S (2004). *In vivo* cancer targeting and imaging with semiconductor quantum dots. Nat. Biotechnol..

[14] Smith AM, Dave S, Nie S, True L, Gao X (2006). Multicolor quantum dots for molecular diagnostics of cancer. Expert Rev. Mol. Diagn..

[15] Freeman R (2012). Optical aptasensors for the analysis of the vascular endothelial growth factor (VEGF). Anal. Chem..

[16] Hu X, Gao X (2010). Silica−Polymer Dual Layer-Encapsulated Quantum Dots with Remarkable Stability. ACS Nano.

[17] Boldt K, Bruns OT, Gaponik N, Eychmüller A (2006). Comparative Examination of the Stability of Semiconductor Quantum Dots in Various Biochemical Buffers. J. Phys. Chem. B.

[18] Ryman-Rasmussen JP, Riviere JE, Monteiro-Riviere NA (2006). Penetration of intact skin by quantum dots with diverse physicochemical properties. Toxicol. Sci..

[19] Jaiswal JK, Mattoussi H, Mauro JM, Simon SM (2003). Long-term multiple color imaging of live cells using quantum dot bioconjugates. Nat. Biotechnol..

[20] Rozenzhak SM (2005). Cellular internalization and targeting of semiconductor quantum dots. Chem. Commun..

[21] Derfus AM, Chan WCW, Bhatia SN (2004). Probing the cytotoxicity of semiconductor quantum dots. Nano Lett..

[22] Bangham AD, Standish MM, Watkins JC (1965). Diffusion of univalent ions across the lamellae of swollen phospholipids. J. Mol. Biol..

[23] Jesorka A, Orwar O (2008). Liposomes: technologies and analytical applications. Annu. Rev. Anal. Chem..

[24] Fielding RM (1991). Liposomal drug delivery. Advantages and limitations from a clinical pharmacokinetics and therapeutic perspective. Clin. Pharmacokinet..

[25] Zylberberg C, Matosevic S (2016). Pharmaceutical liposomal drug delivery: a review of new delivery systems and a look at the regulatory landscape. Drug Deliv..

[26] Gopalakrishnan G (2006). Multifunctional lipid/quantum dot hybrid nanocontainers for controlled targeting of live cells. Angew. Chem. Int. Ed. Engl..

[27] Sigot V, Arndt-Jovin DJ, Jovin TM (2010). Targeted Cellular Delivery of Quantum Dots Loaded on and in Biotinylated Liposomes. Bioconjug. Chem..

[28] Weng KC (2008). Targeted Tumor Cell Internalization and Imaging of Multifunctional Quantum Dot-Conjugated Immunoliposomes *in Vitro* and *in Vivo*. Nano Lett..

[29] Muthu MS, Kulkarni SA, Raju A, Feng S-S (2012). Theranostic liposomes of TPGS coating for targeted co-delivery of docetaxel and quantum dots. Biomaterials.

[30] Ruan G, Agrawal A, Marcus AI, Nie S (2007). Imaging and tracking of Tat peptide-conjugated quantum dots in living cells: New insights into nanoparticle uptake, intracellular transport, and vesicle shedding. J. Am. Chem. Soc..

[31] Dudu V, Ramcharan M, Gilchrist M, Holland E, Vazquez M (2008). Liposome delivery of quantum dots to the cytosol of live cells. J. Nanosci. Nanotechnol..

[32] Al-Jamal WT (2008). Lipid-quantum dot bilayer vesicles enhance tumor cell uptake and retention *in vitro* and *in vivo*. ACS Nano.

[33] Zhang L-W, Wen C-J, Al-Suwayeh SA, Yen T-C, Fang J-Y (2012). Cisplatin and quantum dots encapsulated in liposomes as multifunctional nanocarriers for theranostic use in brain and skin. J. Nanoparticle Res..

[34] Damalakiene L, Karabanovas V, Bagdonas S, Valius M, Rotomskis R (2013). Intracellular distribution of nontargeted quantum dots after natural uptake and microinjection. Int. J. Nanomedicine.

[35] Camblin M (2014). Polymersomes containing quantum dots for cellular imaging. Int. J. Nanomedicine.

[36] Jahn K, Buschmann V, Hille C (2015). Simultaneous Fluorescence and Phosphorescence Lifetime Imaging Microscopy in Living Cells. Sci. Rep..

[37] Techen A, Hille C, Dosche C, Kumke MU (2012). Fluorescence study of drug-carrier interactions in CTAB/PBS buffer model systems. J. Colloid Interface Sci..

[38] Chen C-S, Yao J, Durst RA (2006). Liposome encapsulation of fluorescent nanoparticles: Quantum dots and silica nanoparticles. J. Nanoparticle Res..

[39] Generalov R (2011). Entrapment in phospholipid vesicles quenches photoactivity of quantum dots. Int. J. Nanomedicine.

[40] Han Y, Lee J, Lee Y, Kim SW (2011). Measurement of the diffusion coefficients of fluorescence beads and quantum dots by using fluorescence correlation spectroscopy. J. Korean Phys. Soc..

[41] Zhou J, Wang Q, Zhang C (2013). Liposome–Quantum Dot Complexes Enable Multiplexed Detection of Attomolar DNAs without Target Amplification. J. Am. Chem. Soc..

[42] Dong C, Chowdhury B, Irudayaraj J (2013). Probing site-exclusive binding of aqueous QDs and their organelle-dependent dynamics in live cells by single molecule spectroscopy. Analyst.

[43] Yaghini E (2014). Fluorescence Lifetime Imaging and FRET-Induced Intracellular Redistribution of Tat-Conjugated Quantum Dot Nanoparticles through Interaction with a Phthalocyanine Photosensitiser. Small.

[44] Damalakiene L, Karabanovas V, Bagdonas S, Rotomskis R (2016). Fluorescence-Lifetime Imaging Microscopy for Visualization of Quantum Dots’ Endocytic Pathway. Int. J. Mol. Sci..

[45] Kundrotas G (2019). Uptake and distribution of carboxylated quantum dots in human mesenchymal stem cells: cell growing density matters. J. Nanobiotechnology.

[46] Sagolla K, Löhmannsröben H-G, Hille C (2013). Time-resolved fluorescence microscopy for quantitative Ca^2+^ imaging in living cells. Anal. Bioanal. Chem..

[47] Just F, Walz B (1994). Salivary glands of the cockroach, Periplaneta americana: new data from light and electron microscopy. J. Morphol..

[48] Rech I, Labanca I, Ghioni M, Cova S (2006). Modified single photon counting modules for optimal timing performance. Rev. Sci. Instrum..

[49] Heinze KG, Koltermann A, Schwille P (2000). Simultaneous two-photon excitation of distinct labels for dual-color fluorescence crosscorrelation analysis. Proc. Natl. Acad. Sci. USA.

[50] Petersen NO (1986). Scanning fluorescence correlation spectroscopy. I. Theory and simulation of aggregation measurements. Biophys. J..

[51] Schwille P, Haupts U, Maiti S, Webb WW (1999). Molecular dynamics in living cells observed by fluorescence correlation spectroscopy with one- and two-photon excitation. Biophys. J..

[52] Ries J, Schwille P (2006). Studying slow membrane dynamics with continuous wave scanning fluorescence correlation spectroscopy. Biophys. J..

[53] Ries J, Chiantia S, Schwille P (2009). Accurate determination of membrane dynamics with line-scan FCS. Biophys. J..

[54] Matos ALL, Pereira G, Cabral Filho PE, Santos BS, Fontes A (2017). Delivery of cationic quantum dots using fusogenic liposomes in living cells. J. Photochem. Photobiol. B Biol..

[55] Simberg D, Weisman S, Talmon Y, Barenholz Y (2004). DOTAP (and other cationic lipids): chemistry, biophysics, and transfection. Crit. Rev. Ther. Drug Carrier Syst..

[56] Hwang T-L, Lee W-R, Hua S-C, Fang J-Y (2007). Cisplatin encapsulated in phosphatidylethanolamine liposomes enhances the *in vitro* cytotoxicity and *in vivo* intratumor drug accumulation against melanomas. J. Dermatol. Sci..

[57] Batalla J (2015). Encapsulation efficiency of CdSe/ZnS quantum dots by liposomes determined by thermal lens microscopy. Biomed. Opt. Express.

[58] Müller RH, Jacobs C, Kayser O (2001). Nanosuspensions as particulate drug formulations in therapy. Rationale for development and what we can expect for the future. Adv. Drug Deliv. Rev..

[59] Nguyen TA, Tang QD, Doan DCT, Dang MC (2016). Micro and nano liposome vesicles containing curcumin for a drug delivery system. Adv. Nat. Sci. Nanosci. Nanotechnol..

[60] Ikeda A (2011). Formation and regulation of fullerene-incorporation in liposomes under the phase transition temperature. Org. Biomol. Chem..

[61] Resch-Genger, U., Grabolle, M., Cavaliere-jaricot, S., Nitschke, R. & Nann, T. Quantum dots versus organic dyes as fluorescent labels. *Nat. Methods* **5**, 763–775 (2008). 10.1038/nmeth.1248 18756197

[62] Kulvietis V, Streckyte G, Rotomskis R (2011). Spectroscopic investigations of CdTe quantum dot stability in different aqueous media. Lith. J. Phys..

[63] Petrášek Z, Schwille P (2008). Precise measurement of diffusion coefficients using scanning fluorescence correlation spectroscopy. Biophys. J..

[64] Dertinger T (2007). Two-focus fluorescence correlation spectroscopy: a new tool for accurate and absolute diffusion measurements. ChemPhysChem.

[65] Kuno M, Fromm DP, Hamann HF, Gallagher A, Nesbitt DJ (2000). Nonexponential “blinking” kinetics of single CdSe quantum dots: A universal power law behavior. J. Chem. Phys..

[66] Alves NJ (2013). Functionalized liposome purification via Liposome Extruder Purification (LEP). Analyst.

[67] Walz B, Baumann O, Krach C, Baumann A, Blenau W (2006). The aminergic control of cockroach salivary glands. Arch. Insect Biochem. Physiol..

[68] Lahn M, Dosche C, Hille C (2011). Two-photon microscopy and fluorescence lifetime imaging reveal stimulus-induced intracellular Na^+^ and Cl^−^ changes in cockroach salivary acinar cells. Am. J. Physiol. Cell Physiol..

[69] Berezin MMY, Achilefu S (2011). Fluorescence Lifetime Measurements and Biological Imaging. Chem. Rev..

[70] Niesner R, Peker B, Schlüsche P, Gericke K-H (2004). Noniterative biexponential fluorescence lifetime imaging in the investigation of cellular metabolism by means of NAD(P)H autofluorescence. ChemPhysChem.

[71] Hasinoff BB, Dreher R, Davey JP (1987). The association reaction of yeast alcohol dehydrogenase with coenzyme is partly diffusion-controlled in solvents of increased viscosity. Biochim. Biophys. Acta.

[72] Radoszkowicz L (2011). Time-resolved emission of flavin adenine dinucleotide in water and water–methanol mixtures. Phys. Chem. Chem. Phys..

[73] Brock R, Hink MA, Jovin TM (1998). Fluorescence correlation microscopy of cells in the presence of autofluorescence. Biophys. J..

[74] Mastro AM, Babich MA, Taylor WD, Keith AD (1984). Diffusion of a small molecule in the cytoplasm of mammalian cells. Proc. Natl. Acad. Sci..

[75] Jaiswal JK, Simon SM (2004). Potentials and pitfalls of fluorescent quantum dots for biological imaging. Trends Cell Biol..

[76] Silver J, Ou W (2005). Photoactivation of quantum dot fluorescence following endocytosis. Nano Lett..

[77] Wachsmuth M, Waldeck W, Langowski J (2000). Anomalous diffusion of fluorescent probes inside living cell investigated by spatially-resolved fluorescence correlation spectroscopy. J. Mol. Biol..

[78] Pagano RE, Weinstein JN (1978). Interactions of liposomes with mammalian cells. Annu. Rev. Biophys. Bioeng..

[79] Mislick KA, Baldeschwieler JD (1996). Evidence for the role of proteoglycans in cation-mediated gene transfer. Proc. Natl. Acad. Sci. USA.

[80] Bothun GD, Rabideau AE, Stoner MA (2009). Hepatoma cell uptake of cationic multifluorescent quantum dot liposomes. J. Phys. Chem. B.

[81] Kolašinac R (2018). Deciphering the Functional Composition of Fusogenic Liposomes. Int. J. Mol. Sci..

[82] Karanth H, Murthy RSR (2007). pH-Sensitive liposomes-principle and application in cancer therapy. J. Pharm. Pharmacol..

[83] Martin OC, Pagano RE (1987). Transbilayer movement of fluorescent analogs of phosphatidylserine and phosphatidylethanolamine at the plasma membrane of cultured cells. Evidence for a protein-mediated and ATP-dependent process(es). J. Biol. Chem..

[84] Vu TQ, Lam WY, Hatch EW, Lidke DS (2015). Quantum dots for quantitative imaging: from single molecules to tissue. Cell Tissue Res..

[85] Ghosh Y (2012). New insights into the complexities of shell growth and the strong influence of particle volume in nonblinking ‘giant’ core/shell nanocrystal quantum dots. J. Am. Chem. Soc..

[86] Keller AM (2014). 3-Dimensional tracking of non-blinking ‘giant’ quantum dots in live cells. Adv. Funct. Mater..

[87] Torchilin VP (2005). Recent advances with liposomes as pharmaceutical carriers. Nat. Rev. Drug Discov..

[88] Riaz M (2018). Surface Functionalization and Targeting Strategies of Liposomes in Solid Tumor Therapy: A Review. Int. J. Mol. Sci..

